# Tagging of MADS domain proteins for chromatin immunoprecipitation

**DOI:** 10.1186/1471-2229-7-47

**Published:** 2007-09-14

**Authors:** Stefan de Folter, Susan L Urbanus, Lisette GC van Zuijlen, Kerstin Kaufmann, Gerco C Angenent

**Affiliations:** 1 Business Unit Bioscience, Plant Research International, 6700 AA Wageningen, The Netherlands; 2 Current address- National Laboratory of Genomics for Biodiversity (Langebio), CINVESTAV-IPN, Campus Guanajuato, Apartado Postal 629, 36500 Irapuato, Guanajuato, Mexico

## Abstract

**Background:**

Most transcription factors fulfill their role in complexes and regulate their target genes upon binding to DNA motifs located in upstream regions or introns. To date, knowledge about transcription factor target genes and their corresponding transcription factor binding sites are still very limited. Two related methods that allow *in vivo *identification of transcription factor binding sites are chromatin immunoprecipitation (ChIP) and chromatin affinity purification (ChAP). For ChAP, the protein of interest is tagged with a peptide or protein, which can be used for affinity purification of the protein-DNA complex and hence, the identification of the target gene.

**Results:**

Here, we present the results of experiments aiming at the development of a generic tagging approach for the Arabidopsis MADS domain proteins AGAMOUS, SEPALLATA3, and FRUITFULL. For this, Arabidopsis wild type plants were transformed with constructs containing a MADS-box gene fused to either a double *Strep*-tag^® ^II-FLAG-tag, a triple HA-tag, or an eGFP-tag, all under the control of the constitutive double 35S Cauliflower Mosaic Virus (CaMV) promoter. Strikingly, in all cases, the number of transformants with loss-of-function phenotypes was much larger than those with an overexpression phenotype. Using endogenous promoters in stead of the 35S CaMV resulted in a dramatic reduction in the frequency of loss-of-function phenotypes. Furthermore, pleiotropic defects occasionally caused by an overexpression strategy can be overcome by using the native promoter of the gene. Finally, a ChAP result is presented using GFP antibody on plants carrying a genomic fragment of a MADS-box gene fused to GFP.

**Conclusion:**

This study revealed that MADS-box proteins are very sensitive to fusions with small peptide tags and GFP tags. Furthermore, for the expression of chimeric versions of MADS-box genes it is favorable to use the entire genomic region in frame to the tag of choice. Interestingly, though unexpected, it appears that the use of chimeric versions of MADS-box genes under the control of the strong 35S CaMV promoter is a very efficient method to obtain dominant-negative mutants, either caused by cosuppression or by alteration of the activity of the recombinant protein. Finally, we were able to demonstrate AGAMOUS binding to one of its targets by ChAP.

## Background

During the last 15 years, many studies have been performed aiming at the understanding of MADS-box gene function in plants using loss- and gain-of-function approaches, which resulted in a wealth of information about their role in development [1,2]. Far less is known about how they act at the molecular level, how they bind to DNA motifs (*cis*-elements) and activate down-stream target genes. It has been shown that MADS domain proteins are able to bind to the DNA motif CC(A/T)_6_GG, the so-called CArG-box (reviewed in [3]). This motif has also been found in promoter sequences of a small number of genes that have been annotated as target genes (e.g. [4-7]). Nevertheless, the exact requirements for this DNA motif to be bound by MADS-box transcription factors *in vivo *are still unknown. Therefore, methods for the identification of DNA target sites are needed.

A powerful method to identify target sites is chromatin immunoprecipitation (ChIP), which allows purification of *in vivo *formed complexes of a DNA-binding protein and associated DNA (reviewed in [8]). In short, the method involves the fixation of plant tissue and the isolation of the total protein-DNA mixture, followed by an immunoprecipitation step with an antibody directed against the protein of interest. Next, the DNA can be purified, amplified, and finally identified by sequencing. Alternatively, the amplified DNA can be hybridized to micro arrays containing promoter elements or the entire genome as tiled oligonucleotides (ChIP-chip approach, [9,10]). The identification of target genes from MADS domain proteins by ChIP has been reported recently [5,7,11]. A drawback of ChIP is that for each protein of interest a new specific antibody is required. To overcome this drawback, a protein tagging approach with a general tag could be followed, which we refer to as Chromatin Affinity Purification (ChAP). In this approach, a generic tag is fused to the protein of interest and subsequently used to isolate protein-DNA (or protein-protein) complexes based on affinity purification (reviewed in [12-14]).

In this study we focused on three MADS domain proteins from Arabidopsis, namely AGAMOUS (AG), SEPALLATA3 (SEP3), and FRUITFULL (FUL). AG and SEP3 are both floral organ identity proteins, and based on the ABC model [15], represent C- and E-type proteins, respectively (reviewed in [16]). *AG *is necessary for the formation of stamens and carpels and is expressed in the inner two floral whorls [17]. *SEP3 *is expressed in the inner three whorls and is essential for the formation of petals, stamens and carpels in a redundant mode of action with *SEP1 *and *SEP2 *[18-21]. *FUL *has a function in floral meristem identity (early function) and in fruit development (late function) [22-24], and is expressed in the inflorescence meristem, inflorescence stem, cauline leaves, and in developing ovary walls [25]. Here, we report the expression of these three MADS domain proteins in Arabidopsis fused with different tags and the analysis of the phenotypes obtained. Furthermore, the first result obtained with ChAP using a GFP antibody is presented.

## Results

### Protein tagging vectors for plant expression

**Figure 1:**
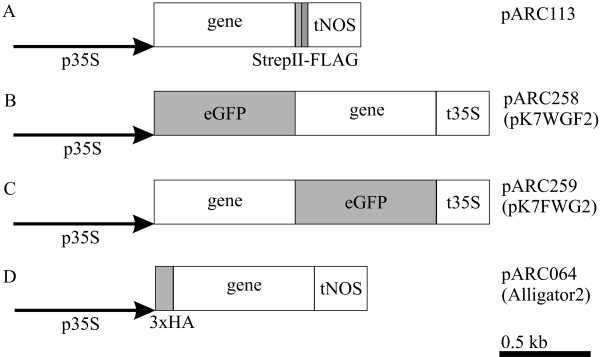
Binary tagging vectors for plant protein expression. (A) C-terminal fusion expression vector with the *Strep*-tag^® ^II and the FLAG-tag. (B) N-terminal fusion expression vector with eGFP. (C) C-terminal fusion expression vector with eGFP. (D) N-terminal fusion expression vector with a triple HA-tag. All vectors contain the constitutive 35S CaMV promoter with the double enhancer for expression.

Four different binary vectors were used for the tagging approach in plants (Figure 1). The first vector (Figure 1A) contains a double tag, the *Strep*-tag^® ^II [26], followed by the FLAG-tag [27], located at the C-terminus of the protein of interest. These peptide tags are both very small, each only 8 amino acids long. Two other vectors (Figure 1B and 1C) contain the coding region for eGFP (enhanced GREEN FLUORESCENT PROTEIN, Clonetech) [28,29], which is either located at the N- or C-terminus of the protein of interest [30]. The fourth vector (Figure 1D) contains a triple HA-tag (hemagglutinin derived) [31], each encoding for a 9 amino acids long peptide. Furthermore, all vectors have a constitutive double 35S CaMV promoter [32,33] to express the fusion products of *AG*, *SEP3*, and *FUL *in transgenic Arabidopsis plants.

### Phenotypic and expression analyses of Arabidopsis lines expressing chimeric MADS-box versions

**Figure 2:**
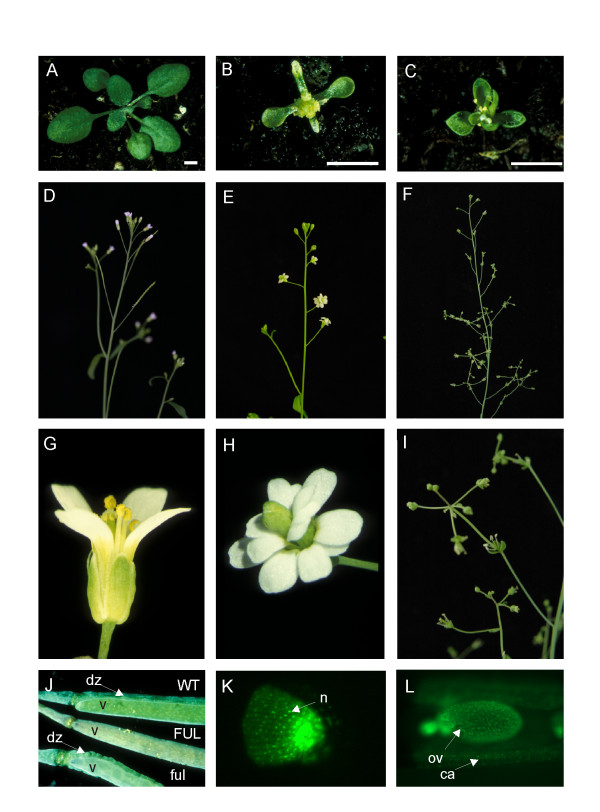
Phenotypes of transgenic Arabidopsis plants with different tagging constructs. (A) Wild-type Arabidopsis at the rosette stage, (D) at the inflorescence stage, and (G) a close-up of a flower. (B) Line with *AG-eGFP *fusion construct showing an *AG *overexpression phenotype (pARC276). (C) Line with *SEP3-eGFP *fusion construct showing a *SEP3 *overexpression phenotype (pARC277). Rosette stage images (A-C) were taken from plants grown under the same conditions and were of the same age (bar indicates relative size). (E, H) Line with *eGFP-AG *fusion construct showing an *ag *mutant phenotype (pARC308). (F, I) Line with *eGFP-SEP3 *fusion construct showing a partial *sep*-like mutant phenotype (pARC309). (J) Siliques of lines with *GFP-FUL *fusion construct with either a *FUL *overexpression (*FUL*), *ful *mutant (*ful*) phenotype, or wild-type phenotype (WT) (pARC310). (K) Arabidopsis root tip and (L) open silique with an ovule of a line expressing *GFP-FUL *fusion construct (pARC310) observed by fluorescence microscopy. dz, dehiscence zone; v, valve; ov, ovule; n, nuclues; ca, carpel wall.

All constructs were introduced into Arabidopsis wild type plants, ecotype Columbia-0, and the transformants obtained were analyzed for overexpression phenotypes. The results are summarized in Table 1 and Figure 2. The expected overexpression phenotypes for *AG *are homeotic changes of floral organs, resembling an *apetala2*-like flower, curly leaves, and early flowering as described by [34]. For ectopic *SEP3 *expression, curly leaves and early flowering are characteristics to be expected [35], while ectopic expression of *FUL *results in siliques that fail to shatter, because the dehiscence zone is absent [23,24].

**Table 1:** Summary of tagged MADS domain proteins in Arabidopsis plants with the observed phenotypes

Construct	Expression cassette	Plants (n)	Phenotypes (%)		
			
			OE	LOF	WT
pARC117	*35S:FUL:StrepII-FLAG:tNOS*	21	-	57	43
pARC118	*35S:AG:StrepII-FLAG:tNOS*	14	-	29	71
pARC276	*35S:AG:GFP:t35S*	42	12	88	-
pARC277	*35S:SEP3:GFP:t35S*	60	8	-	92
pARC308	*35S:GFP:AG:t35S*	54	7	93	-
pARC309	*35S:GFP:SEP3:t35S*	46	-	100	-
pARC310	*35S:GFP:FUL:t35S*	49	10	90	-
pARC346	*35S:3xHA:AG:tNOS*	12	-	50	50
pARC347	*35S:3xHA:SEP3:tNOS*	15	-	-	100
pARC348	*35S:3xHA:FUL:tNOS*	16	-	38	62
pARC422	*gAG:GFP:tNOS*	25	-	20	80
pARC423	*gSEP3:GFP:tNOS*	46	-	-	100
pARC424	*gFUL:GFP:tNOS*	18	-	28	72

n, number of plants; %, percentage of plants; OE, overexpression; LOF, loss-of-function phenotype; WT, wild-type.

**Figure 3:**
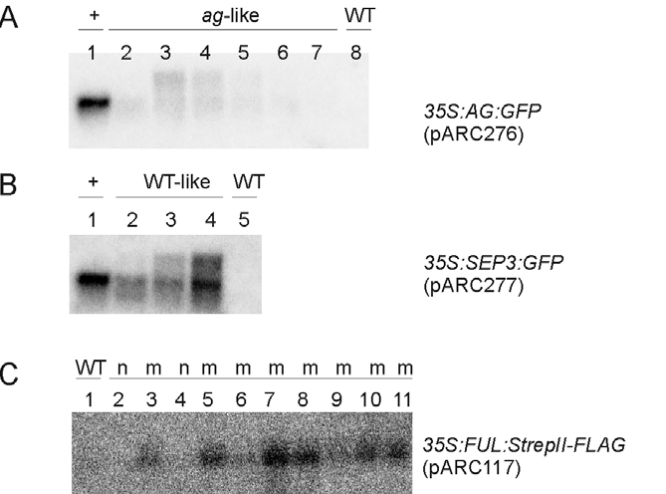
Northern blot analysis of leaf tissue of different Arabidopsis lines containing various tagging constructs. (A) Expression analysis of *AG-eGFP *(pARC276) lines. (B) Expression analysis of *SEP3-eGFP *(pARC277) lines. (C) Expression analysis of *FUL*-*Strep*-tag^® ^II-FLAG-tag (pARC117) lines, *ful*-like plants are indicated with 'm' and WT-like plants with 'n'. WT, wild-type; +, line with an overexpression phenotype.

Overexpression phenotypes were only observed in about 10% of the plants when the eGFP protein was fused either N- or C-terminally (Figure 2B, C, and 2J). Surprisingly, many plants containing an *eGFP *fusion construct revealed a mutant phenotype (Figure 2E, F,H, I, and 2J). Plants with either an overexpression phenotype or a mutant phenotype, obtained with construct pARC276 and pARC277 (Table 1), were analyzed by northern blot hybridization for the expression of the introduced *AG *or *SEP3 *transgenes, respectively (Figures 3A and 3B). This revealed a perfect linkage between plants with an overexpression phenotype having a high ectopic gene expression in leaves, while plants with an *ag *mutant phenotype (pARC276), showed no expression. In stead, the latter plants exhibit a smear in the Northern blot, which is often observed when a gene is cosuppressed [36,37]. Remarkably, for plants containing the *SEP3 *fusion construct (pARC277), no loss-of-function phenotypes were observed, though, the Northern blot showed hallmarks of cosuppression, suggesting that silencing of *SEP3 *may have occurred. Most likely, the paralogs and redundant genes *SEP1 *and *SEP2 *are not affected, which explains that no mutant phenotype was obtained. Plants carrying the *FUL *fusion construct (pARC310) were not molecularly analyzed, but mutant-like plants in a range of severity were observed, which suggest that also cosuppression had occurred. Furthermore, a few overexpression and mutant plants with the *AG*, *SEP3*, and *FUL *fused to *eGFP *were analyzed for fluorescence (Figures 2K and 2L) and confirmed the same linkage between expression and phenotype.

Plants transformed with constructs containing either the *Strep*-tag^® ^II-FLAG-tag or the triple HA-tag displayed only a wild-type- or mutant phenotype. Transgenic plants with construct pARC117, containing the double *Strep*-tag^® ^II-FLAG-tag, were also analyzed by Northern blot for the expression of the *FUL *fusion product (Figure 3C). Remarkably, in contrast to the *eGFP *fusion constructs, all plants with a loss-of-function phenotype revealed ectopic *FUL *expression, which was lacking in plants with a wild-type phenotype. This suggests that this mutant phenotype obtained with the double tag *Strep*-tag^® ^II-FLAG-tag is caused by a dominant-negative effect and not by a cosuppression mechanism.

The plants with the triple HA-tag fusion constructs were analyzed by RT-PCR (data not shown). Plants with a mutant phenotype reminiscent with *ag *(pARC346) or *ful *(pARC348) mutants revealed either no expression, suggesting cosuppression, or overexpression, suggesting a dominant-negative effect, respectively.

### Expression analysis of the SEP3 promoter in Arabidopsis

The constitutive and strong double 35S CaMV promoter resulted in high expression of the transgene in those plants that showed an overexpression phenotype. However, in the case of *AG *and *SEP3*, this promoter caused pleiotropic defects resulting in extremely small and early flowering plants with only a few flowers were produced (Figures 2B and 2C). To overcome this problem, the double 35S CaMV promoter was replaced by the endogenous promoter. A 2.6 kb fragment upstream the ATG start codon of *SEP3 *was fused to the β-glucuronidase reporter gene, encoding for GUS [38]. GUS staining in transgenic Arabidopsis plants was detected in the three inner whorls of the flower (Additional file 1), where *SEP3 *is normally expressed [20]. However, GUS signal was also detected in the sepals, pedicels, and even in cauline and rosette leaves (Additional file 1), suggesting that the upstream region of *SEP3 *is lacking *cis*-acting regulatory regions for correct expression.

**Figure 4:**
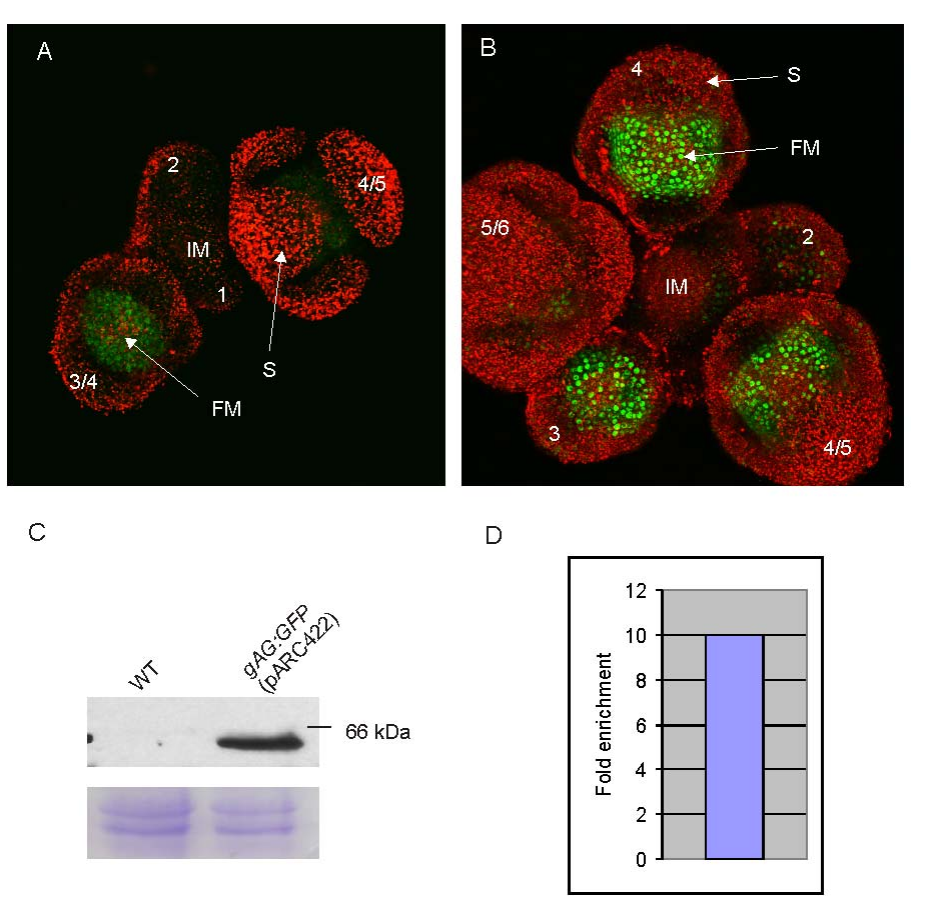
AG and SEP3 expression analysis and chromatin immunoprecipitation (ChIP). Confocal Scanning Laser Microscopical (CSLM) imaging of (A) *gAG:GFP *(pARC422) and (B) *gSEP3:GFP *(pARC423) in the inflorescence. Top view (A, B) of an inflorescence with different floral bud stages (indicated by numbers). The GFP expression (green signal) is predominantly localized in the nuclei of floral meristem cells of flower buds from stage 3 onwards (comprising whorl 3 and 4 for AG, and whorl 2, 3, and 4 for SEP3, respectively). Autofluorescence is visible as red signal. (C) Anti-GFP Western blot with material from Arabidopsis WT and *gAG:GFP *(pARC422) plants. Protein product is detectable in transgenic plants only. Bottom panel shows the Coomassie stained gel serving as loading control. (D) Enrichment of AG target DNA after ChAP with GFP antibody and compared with pre-immune. Quantification of target DNA was done by Real-time PCR using primers corresponding to sequences in the second intron of *AG*. FM, floral meristem, S, sepal, IM, inflorescence meristem, WT, wild-type.

Similar misexpression was observed for the MADS-box genes *AG *and *SEEDSTICK *(*STK*), when only the DNA region upstream the first intron or the ATG, respectively, was fused to the GUS reporter gene [39,40]. In the case of *AG*, it appeared that the second intron, which contains various *cis*-acting regulatory elements [39,41-43] was essential for the right spatial expression pattern, while for correct *STK *expression, the first intron should be included in the reporter constructs [40]. When the *SEP3 *first intron sequence was analyzed in detail different motifs were identified that might act as *cis*-regulatory elements, including a perfect CArG-box (data not shown). To investigate the importance of the *SEP3 *intron sequences, a 3.5 kb genomic fragment of *SEP3*, including upstream and intron sequences, was fused to a GFP tag (pARC423) and introduced into Arabidopsis plants. In contrast to the observed misexpression when only the *SEP3 *upstream region was used, correct spatial and temporal expression was obtained when also the *SEP3 *intron sequences were included (Figure 4). The *gSEP3:GFP *(pARC423) expression is predominantly visible in the nuclei of the floral meristem cells of floral buds from stage 3 onwards (comprising whorl 2, 3, and 4), while there is no or minimal expression in the rest of the inflorescence (Figure 4B). Noteworthy, the number of observed loss-of-function phenotypes with an endogenous MADS-box gene promoter (pARC422 and pARC424) is dramatically less than in the case with the 35S CaMV promoter (Table 1) or even absent in the case of *SEP3 *(pARC423) (Table 1).

In summary, the reported results with *AG *and *STK *and our results with *SEP3 *indicate that intron regions in MADS domain genes are important for correct spatial and temporal expression.

### AG protein detection and chromatin affinity purification

To investigate whether the ChAP procedure using tags is feasible we used transgenic Arabidopsis plants expressing *gAG:GFP *(pARC422) as example. Correct spatial and temporal AG expression was observed, predominantly in the nuclei of the floral meristem cells of floral buds from stage 3 onwards (comprising whorl 3 and 4) (Figure 4A).

First, we analyzed the *gAG:GFP *(pARC422) plants by Western blotting to see whether the chimeric AG protein is detectable with a polyclonal GFP antibody. For this, protein was isolated from nuclei extracts from wild type Arabidopsis (Col-0) plants and compared with extracts from *gAG:GFP *plants. The Western blot (Figure 4C) shows a specific band of the expected size in the *gAG:GFP *plants, which was not present in wild type plants.

Finally, a chromatin affinity purification with a GFP antibody was performed on a protein extract derived from *gAG:GFP *(pARC422) plants. As reported before, AG protein is able to bind to its own intron sequence for autoregulation [7]. This regulatory region was analyzed for enrichment by Real-time PCR, which would demonstrate that the chimeric AG protein is able to bind *in vivo *to its target sequence. The target DNA sequence (*AG *second intron) was 10 fold enriched after affinity purification with GFP antibody demonstrating that chimeric AG is indeed able to bind to its regulatory region (Figure 4D).

## Discussion

The use of epitope tags can facilitate the isolation of protein-DNA or protein-protein complexes. Here, we report a first attempt of employing a generic tagging approach for the MADS domain proteins AG, SEP3, and FUL. Different tags and a combination of tags were used to produce fusion products expressed in plants. There are two important criteria before further steps can be undertaken to identify target genes by Chromatin Affinity Purification (ChAP). The first basic and most important aspect is to obtain stable expression of the fusion protein. Secondly, an expressed fusion protein should be biologically active. Both aspects appeared not to be straight forward and appeared to be dependent on the tags used.

The expression experiments in plants using the constitutive and strong 35S CaMV promoter resulted in mutant phenotypes with all constructs, though, in many cases, not the expected overexpression phenotypes. Remarkably, the percentage of loss-of-function phenotypes obtained was very high, even up to 100% in the case of *GFP:SEP3 *(pARC309). The loss-of-function phenotypes were most likely caused by two phenomena, either by cosuppression in the case of the *eGFP *fusions, or by a dominant-negative effect in the case of the *Strep*-tag^® ^II-FLAG-tag fusions. With the triple HA-tag both phenomena could have happened. These different tags have been used in many organisms and with many different proteins (e.g. [13,31,44-46]), however, it has never been reported that they cause these severe problems related to mRNA expression or activity of a recombinant protein. The high frequency of silencing with the *eGFP *fusions could be related to the 35S CaMV promoter, causing high expression of the transgene. Expression of MADS-box cDNAs under the control of the 35S CaMV promoter without the GFP tag (e.g. [47]) or expression of GFP tags using endogenous MADS-box gene promoters did not reveal such high percentages of cosuppression plants (Table 1), indicating that the combination of 35S CaMV promoter and the GFP tag may induce silencing. The silencing efficiencies of MADS-box gene expression using the GFP tag in combination with the 35S CaMV promoter appeared to be comparable when using an RNA interference strategy [48]. The only exception on this rule is *SEP3:GFP *(pARC277), which did not result in any plant with a loss-of-function phenotype. In contrast, all *GFP:SEP3 *plants show a mutant phenotype. Although an intriguing observation, an explanation is missing. The altered biological activity of the FUL protein fused to short peptide tags, here referred to as 'dominant-negative' mode of action, could be caused by either trapping interacting proteins and forming non-functional protein complexes, steric hindrance preventing certain interactions, or altered folding of the protein. However, functionality of a fusion product with an epitope tag has to be analyzed case by case. It depends on the tag used and the effect it may have on the protein of interest. Our results indicate that the activity of MADS-box genes and their products can be dramatically affected by fusions with small peptide tags and GFP tags at both N- and C-termini. This high sensitivity to fusions, however, can also be used as an effective method to obtain high percentages of dominant loss-of-function mutants.

A drawback of an overexpression strategy could be the occurrence of unwanted pleiotropic effects, e.g. early flowering or a reduced number of flowers. Furthermore, overexpression or ectopic expression does not mimic the natural situation. The most elegant solution is to express the genes under their native promoter in a mutant background, which will directly reveal their biological activity and eliminate any competition with the untagged endogenous protein. For the isolation of the native promoter, often DNA sequences upstream the ATG start codon are cloned, although no general rules are available that can predict the promoter region (reviewed in [49]). This approach was followed for the *SEP3 *promoter, however, it revealed a lack of specificity compared to previously reported *in situ *hybridization experiments [20]. As described previously for the MADS-box genes *AG *and *STK*, intron sequences are important for correct expression [39,40]. This appears also to be the case for *SEP3*, because fusion of *GFP *to a 3.5 kb genomic fragment of *SEP3 *including upstream and intron sequences revealed correct expression patterns. Finally and most importantly, it appeared possible to perform ChAP using a GFP antibody on plants that carried a genomic *AG *fragment (including upstream and intron sequences) fused to GFP (pARC422).

## Conclusion

A powerful method to identify target genes is ChIP or related ChAP. ChAP makes use of an epitope tag fused to the protein of interest and this study revealed that the activities of MADS-box proteins are very sensitive to fusions with small peptide and GFP tags. Furthermore, for the expression of chimeric versions of MADS-box genes it is favorable to use the entire genomic region in frame with the tag of choice. Interestingly, though unexpected, it appears that the use of chimeric versions of MADS-box genes under the control of the strong 35S CaMV promoter is a very effective method to obtain loss-of-function mutants, either caused by cosuppression or by alteration of the activity of the recombinant protein. Finally, ChAP is possible with a chimeric MADS-box protein using a GFP antibody.

## Methods

### Plant growth

*Arabidopsis thaliana*, ecotype Columbia-0 (Col-0) plants were grown under normal greenhouse or growth chamber conditions (22°C, long day light regime).

### Construction of binary vectors and plant transformation

The vector with the C-terminal double tag *Strep*-tag^® ^II (WSHPQFEK) and the FLAG-tag (DYKDDDDK) is called pARC113. The double tag is constructed with two forward and three reverse complementary primers resulting in, with Arabidopsis codon usage, 5'-CTCGAGTGGTCTCATCCTCAATTTGAAAAGTCTTCTGATTACAAGGATGATGATGATAAGTAACTCGAG-3' (nucleotides coding for the tags are underlined). Between the two tags are two serine amino acid residues functioning as linker and after the FLAG-tag a stop codon is introduced. In brief, 1 ul of each primer (100 pmol/ul) were pooled together, incubated for 10 min at 96°C and slowly cooled down to room temperature to create double stranded fragments. The fragments were phosphorylated with 2 ul T4 kinase (10 U/ul) and incubated for 30 min at 37°C. Next, the 69 nucleotides double stranded fragments were isolated from a 12% polyacrylamide gel. Subsequently, the fragment was cloned into an *Xho*I digested binary pGD121 vector [50], containing a double 35S CaMV promoter (derived from pGD120; [51]). Full length open reading frames for *AG *(At4g18960; encoding 252 amino acids), *SEP3 *(At1g24260; encoding 251 amino acids), and *FUL *(At5g60910; encoding 242 amino acids) were amplified with gene specific primers from the start to the stop codon, clones for C-terminal fusions lack the stop codon, and were subcloned in pGEM-T^® ^Easy (Promega, Madison, WI) and/or subcloned with the Gateway™ Technology (Invitrogen, Carlsbad, CA). After sequence control, all genes were cloned (in pARC113) and/or recombined (in pARC064, pARC258, and pARC259) in the appropriate vectors to make the fusion constructs.

A 2.6 kb *SEP3 *region upstream of the ATG was amplified with specific primers (PRO117 5'-CACCGGCGCGCCATCCATCCATCCAAATGGGACC-3' and PRO118 5'-GAAGCTTTTTCTTTTTCTTTCTCCTCTCCC-3') and recombined with the Gateway™ Technology in pENTR/D-TOPO (Invitrogen), followed by recombination in the binary vector pBGWFS7 [30], resulting in a transcriptional *eGFP-GUS *fusion construct (pARC213).

Genomic fragments for *AG *(6882 bp), *SEP3 *(3489 bp), and *FUL *(5298 bp) were amplified with gene specific primers, a forward primer located in the upstream region, PRO433 *AG*-5'- CACCGATCAAAGACTACACATCAC-3', PRO407 *SEP3*-5'- CACCCATACCTTTGTGTCCATCAC-3', and PRO429 *FUL*-5'- CACCTCGATCAGAATTTGAGCTG-3', and a reverse primer in the 3'-region lacking the stop codon for each gene, PRO431 *AG*-5'-CACTAACTGGAGAGCGGTTTG-3', PRO408 *SEP3*-5'- AATAGAGTTGGTGTCATAAGGTAACC-3', and PRO430 *FUL*-5'- CTCGTTCGTAGTGGTAGGAC-3', and recombined in pENTR/D-TOPO. After sequence control, all genomic fragments were recombined in the binary vector pMDC204 [52], resulting in translational GFP6 fusion constructs (pARC422, pARC423, and pARC424, respectively).

Arabidopsis plants were transformed with *Agrobacterium tumefaciens *strain GV3101 using the floral dip method [53].

### RNA gel blot analysis

Total RNA was isolated from frozen plant tissue with the RNeasy plant RNA extraction kit (Qiagen). Five micrograms of each RNA sample was denaturated by 1.5 M glyoxal, separated on a 1.2% agarose gel in 15 mM Na-phosphate buffer pH 6.5, checked for equal loading, and followed by blotting onto Hybond-N + membrane (Amersham Biosciences, Piscataway, NJ) in 25 mM Na-phosphate buffer pH 6.5. Probes were labeled with the RadPrime DNA Labeling System (Invitrogen) and blots were hybridized as described by Angenent et al. (1992) [54]. Gene specific probes were amplified by PCR with the following primers: PRO383 *AG*-5'-GGGTCAATGTCTCCCAAAGA-3' and PRO384 *AG*-5'-CTAACTGGAGAGCGGTTTGG-3', PRO105 *SEP3*-5'-GTCTAGAATGGGAAGAGGGAGAGTAG-3' and PRO106 *SEP3*-5'-CGGATCCAATAGAGTTGGTGTCATAAGGTAACC-3'. The *FUL *fragment was derived from a pGEM-T^® ^Easy (Promega) clone digested with *Xba*I-*Kpn*I.

### GUS assay

To detect β-glucuronidase (GUS) activity [38], plant tissue was fixed in 90% ice-cold acetone for 1 h at -20°C, followed by three rinses with 0.1 M Na-phosphate buffer pH 7.0 containing 1 mM potassium ferrocyanide. The three rinse steps in total took 1 h and during the first rinse step vacuum was applied for ~ 15 min. Finally, the substrate was added to the samples, containing 50 mM Na-phosphate buffer pH 7.0, 1 mM EDTA, 0.1% (v/v) Triton ×-100, 1 mM potassium ferrocyanide, and 1 mM X-Gluc (Duchefa, Haarlem, The Netherlands), and vacuum was applied for 5 min, followed by overnight incubation at 37°C in the dark. Chlorophyll was removed by, first, 1 h incubation in 96% ethanol and then transference to 70% ethanol.

### Microscopy

Plant tissue was observed for GFP expression with a Zeiss Axioskop UV-microscope, equipped with filter set 13 (excitation BP 470/20, beamsplitter FT 493, emission BP 505–530). Images were taken with a Leica DFC320 digital camera and an exposure time of 18 seconds was used. Confocal Scanning Laser Microscopical (CSLM) imaging of plant tissue was performed with a Zeiss LSM 510 inverted confocal microscope using a 40× C-Apochromat (NA 1,2 W Korr) lens. The tissue was embedded in the wells of a Silicone Isolator (Grace Bio-Labs, Bend, OR) with 0.8% agar 0.5× MS. GFP was excited with the 488 line of an argon ion laser. The emission of GFP was filtered with a 505–530 nm bandpassfilter, while the red autofluorescence of the plant tissue was filtered with a 650 nm long-pass filter. 3D projections of the obtained confocal z-stacks were made with the Zeiss LSM Image Browser Version 4.

### Chromatin Affinity Purification (ChAP)

The procedure was performed as previously described [7] with some modifications. Fixed (15–30 min) inflorescence tissue (~ 0.8 g) was used of transgenic Arabidopsis plants containing construct pARC422 that carries a genomic *AG *fragment fused with GFP. Chromatin was solubilized on ice with a probe sonicator (MSE, Soniprep 150) by 3 cycles of 15 sec pulses of half maximal power with 30 sec cooling time between pulses. GFP antibody was used for the affinity purification (ab290; Abcam, Cambridge, UK) and for the negative control complete rabbit serum. For pre-clearing and affinity purification Protein A-Agarose beads were used (sc-2001; Santa Cruz Biotechnology, Santa Cruz, CA). After elution of the beads, samples were treated with proteinase K, followed by precipitation. The precipitated DNA was dissolved in 100 ul water, purified with a PCR purification kit (Qiagen, Valencia, CA), and eluted with 30 ul EB (water containing 10 mM Tris, pH 8). Enrichment of the target region was determined using a real-time PCR detection system (MyiQ, Bio-Rad Laboratories, Hercules, CA) by comparing the affinity purified sample (anti-GFP) with the negative control (rabbit serum). The results between the two samples were normalized using sequences of *Heat Shock Factor1 *(*HSF1*; At4g17750). The following primers were used, PRO469 *AG*-5'- TGGTCTGCCTTCTACGATCC-3' and PRO470 *AG*-5'-CAACAACCCATTAACACATTGG-3', PDS1045 *HSF1*-5'- GCTATCCACAGGTTAGATAAAGGAG-3' and PDS1046 *HSF1*-5'- GAGAAAGATTGTGTGAGAATGAAA-3'.

### Protein isolation and detection

Nuclei extraction from 0.5 g of Arabidopsis inflorescences was performed according to the protocol used for ChIP experiments [7]. The nuclei pellet was resuspended in 120 ul 2× SDS sample buffer, incubated on ice and centrifuged at 20800 × g for 10 min at 4°C. The supernatant was boiled for 5 min. Western blotting was performed essentially as described previously [55]. The GFP antibody (ab290; Abcam) was used in a 1:5000 dilution.

## Competing interests

The author(s) declares that there are no competing interests. 

## Authors' contributions

SdF and GA conceived and designed the experiments. SdF, SU, KK, and LvZ carried out the experiments. SdF and GA drafted the manuscript. All authors read and approved the final manuscript.

## Supplementary Material

Additional file 1 *SEP3 *expression analysis in transgenic Arabidopsis plants. (A-C) GUS expression patterns of *SEP3 *promoter *GUS *fusion (pARC213) in different tissues, (A) inflorescence, (B) silique, and (C) rosette leaf. ov, ovule. Click here for file
