# Supplementary figures and images for: Tagging of MADS domain proteins for chromatin immunoprecipitation
Source: BMC Plant Biol. 2007 Sep 14;7:47. doi: 10.1186/1471-2229-7-47 (PMC2071916; doi:10.1186/1471-2229-7-47)

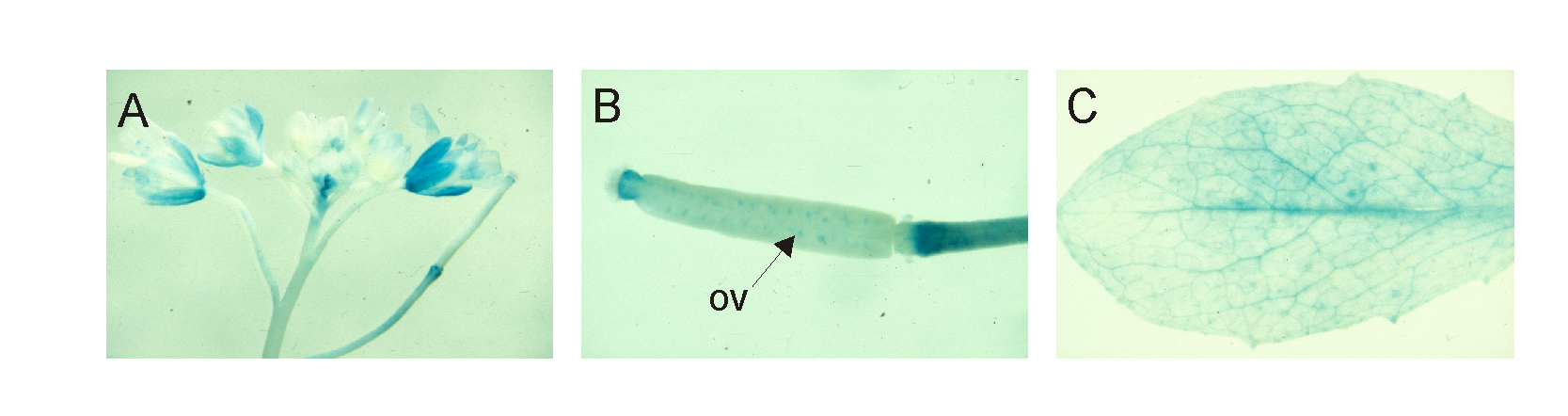

Supplement: Additional file 1 — SEP3 expression analysis in transgenic Arabidopsis plants. (A-C) GUS expression patterns of SEP3 promoter GUS fusion (pARC213) in different tissues, (A) inflorescence, (B) silique, and (C) rosette leaf. ov, ovule. [file 1471-2229-7-47-S1.png]
